# Requirement for Chloride Channel Function during the Hepatitis C Virus Life Cycle

**DOI:** 10.1128/JVI.02946-14

**Published:** 2015-01-21

**Authors:** Zsofia Igloi, Bjorn-Patrick Mohl, Jonathan D. Lippiat, Mark Harris, Jamel Mankouri

**Affiliations:** a School of Molecular and Cellular Biology, Faculty of Biological Sciences, University of Leeds, Leeds, United Kingdom; b School of Biomedical Sciences, Faculty of Biological Sciences, University of Leeds, Leeds, United Kingdom

## Abstract

Hepatocytes express an array of plasma membrane and intracellular ion channels, yet their role during the hepatitis C virus (HCV) life cycle remains largely undefined. Here, we show that HCV increases intracellular hepatic chloride (Cl^−^) influx that can be inhibited by selective Cl^−^ channel blockers. Through pharmacological and small interfering RNA (siRNA)-mediated silencing, we demonstrate that Cl^−^ channel inhibition is detrimental to HCV replication. This represents the first observation of the involvement of Cl^−^ channels during the HCV life cycle.

## TEXT

Hepatitis C virus (HCV) is a major human pathogen that causes chronic liver disease, including hepatocellular carcinoma and cirrhosis (1). Between 130 and 170 million people are now chronically infected, and HCV-related disease leads to 350,000 deaths per year (2). Recent developments have led to the discovery of new direct-acting antivirals (DAA) (3), including the HCV polymerase (NS5B) inhibitor sofosbuvir. While these DAAs signify the encouraging progress of HCV treatment regimens, issues of their high cost coupled with potential resistance remain. Thus, research into new antiviral targets is still required (4).

HCV is an enveloped virus with a positive-sense, single-stranded RNA genome belonging to the genus Hepacivirus within the Flaviviridae family (5, 6). The 9.6-kb HCV genome encodes a single large polyprotein of 3,000 amino acids, which is processed by viral and host cellular proteases into 10 functional proteins (6, 7). These include the structural proteins (core, E1, E2) and seven nonstructural proteins (p7, NS2, NS3, NS4A, NS4B, NS5A, NS5B) (6).

HCV displays tropism primarily for hepatocytes, the parenchymal cells of the liver (6, 8). Hepatocytes are multifunctional epithelial cells that engage in transcellular solute transport, processing of metabolites, and the synthesis and secretion of numerous important proteins. In common with all eukaryotic cells, hepatocytes possess ion channels at the plasma membrane and in multiple intracellular compartments (9, 10). We have previously shown that HCV NS5A can inhibit a hepatic proapoptotic host cell K^+^ channel (Kv2.1, *KCNB1*) to maintain the survival of infected cells (10) by perturbing signaling leading to Kv2.1 activation (11). There are over 230 genes encoding ion channel subunits in the human genome (12), but no further functional role of these channels during HCV pathogenesis has been assigned. We therefore assessed the effects of modulating all the major cellular ion channel families on the HCV life cycle.

**FIG 1:**
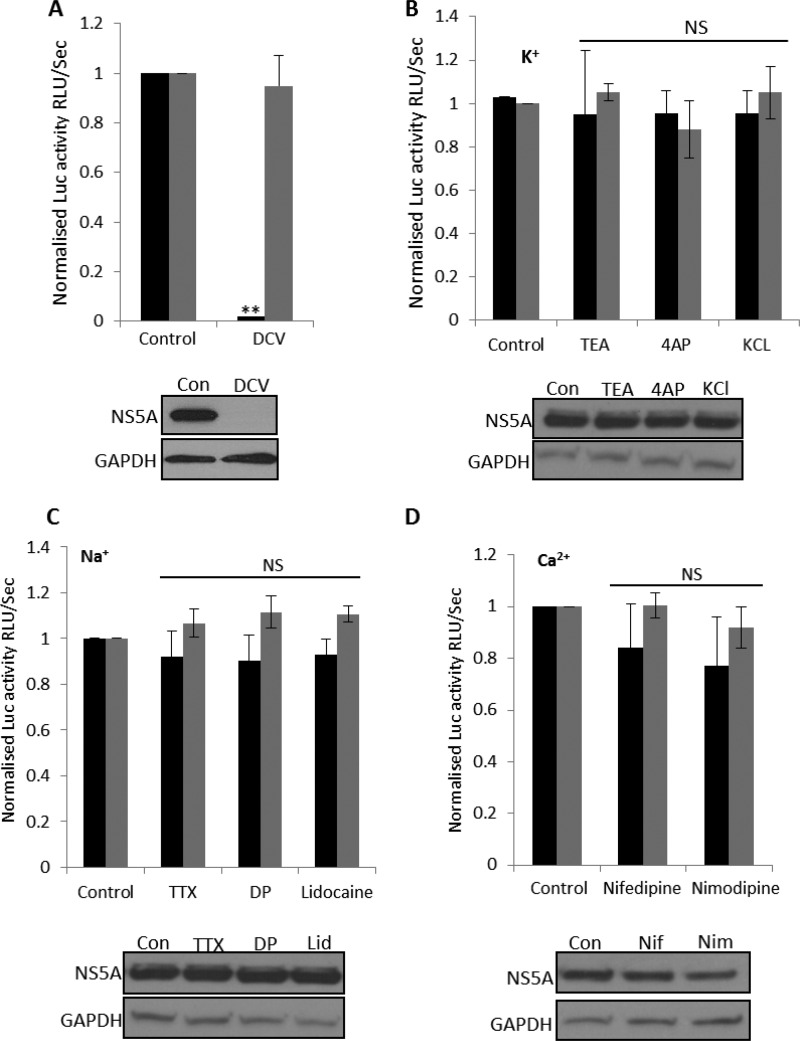
Assessment of ion channel modulators on HCV replication. SGR–Feo–JFH-1 cells stably expressing a luciferase subgenomic replicon (genotype 2a) were treated with DCV (1 μM) (A) or the indicated K^+^ (10 mM TEA, 40 mM KCl, 1 mM 4AP) (B), Na^+^ (5 μM TTX, 100 μM disopyramide phosphate [DP], 1 mM lidocaine) (C), or Ca^2+^ (5 μM nifedipine, 5 μM nimodipine) (D) channel modulators for 24 h. Luciferase activity (relative luminescence units [RLU] per second) was used as a measure of genome replication (black bars), and cell viability was assessed by 3-(4,5-dimethyl-2-thiazolyl)-2,5-diphenyl-2H-tetrazolium bromide (MTT) assays (gray bars). Results were calculated relative to those for an untreated control. Error bars represent the standard errors of the means (SEM) of results from three independent experiments. **, significant difference from the control value (*P* < 0.05); NS, no differences at a significance level of 0.05. Immunoblots of cell lysates 48 h after the drug treatment were analyzed with polyclonal anti-NS5A antiserum or anti-GAPDH (glyceraldehyde-3-phosphate dehydrogenase; loading control). Representative Western blots are shown below each corresponding graph. Con, control; Lid, lidocaine; Nif, nifedipine; Nim, nimodipine.

To determine if the activities of cellular ion channels are required during the HCV life cycle, we first assessed virus genome replication using the bicistronic JFH-1 genotype 2a subgenomic replicon (SGR), which expresses a firefly luciferase-neomycin phosphotransferase fusion protein (SGR–Feo–JFH-1) (13). A cell line stably harboring this SGR was treated with compounds previously characterized as modulating specific ion channel families. Replication was monitored through the measurement of luciferase activity and confirmed by Western blotting for NS5A. Daclatasvir (DCV) was included as a known inhibitor of virus replication (14); it reduced luciferase activity by 94% ± 5% (Fig. 1A) and reduced NS5A expression to undetectable levels. As shown in Fig. 1B to D, there were no effects on luciferase activity or NS5A expression when cells were treated with tetraethylammonium (TEA), a broadly acting blocker of potassium (K^+^) channels (15), 4-aminopyridine (4AP), a blocker of voltage-gated K^+^ channels (16), or KCl to collapse K^+^ gradients. Similarly, when cells were treated with blockers of voltage-gated Na^+^ channels (NaV), tetrodotoxin (TTX), disopyramide phosphate (DP), and lidocaine (17, 18) or with inhibitors of plasma membrane Ca^2+^ channels, namely, nifedipine (Nif) and nimodipine (Nim) (19), no reduction in genome replication 24 h after compound treatment occurred (Fig. 1B to D). We thus concluded that the inhibition of K^+^, Na^+^, and Ca^2+^ channels does not have an impact on HCV genome replication.

**FIG 2:**
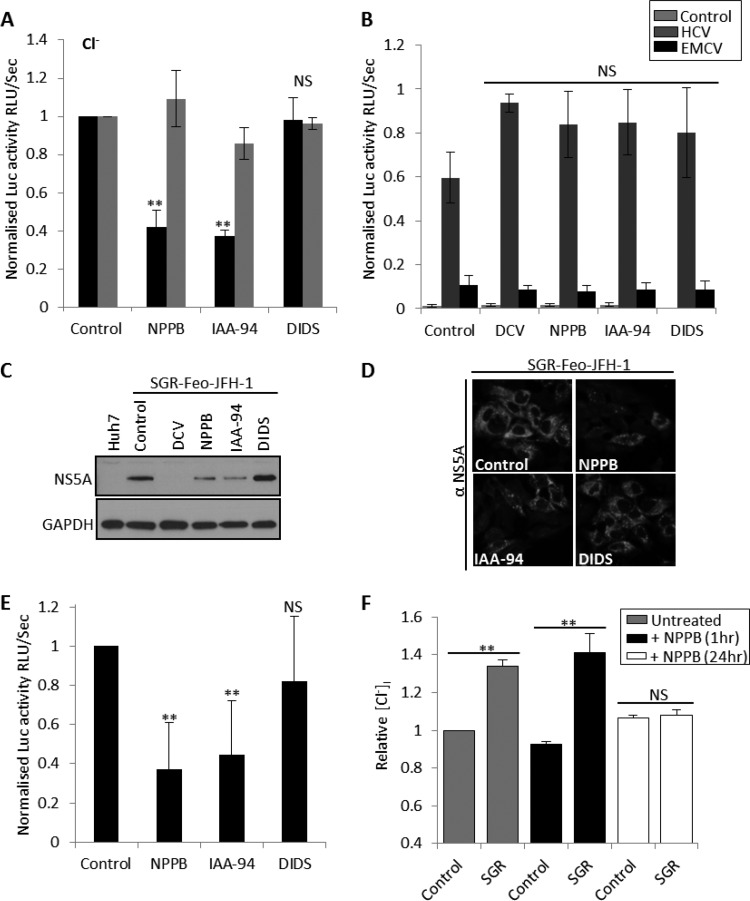
Assessment of the role of Cl^−^ channels during HCV replication. (A) JFH-1–SGR-Feo cells were treated with the Cl^−^ channel inhibitors NPPB (10 μM), IAA-94 (100 μM), and DIDS (100 μM) for 24 h, and luciferase expression was assessed as described for Fig. 1. Results were calculated relative to those for an untreated control. Error bars represent the SEM from three independent experiments. **, significant difference from the value for the untreated control (*P* < 0.05); NS, no differences at the 0.05 significance level. (B) Huh7 cells were transfected with either the pRZF vector (mock control) or the pRZF vector containing the firefly luciferase gene under the translational control of the HCV or the encephalomyocarditis virus (EMCV) IRES and the promoter-driven Renilla luciferase gene as previously described (10). Four hours posttransfection, cells were treated with DCV, IAA-94, NPPB, and DIDS for 48 h, and luciferase expression was assessed as described for Fig. 1. Error bars represent the SEM from three independent experiments. Values are normalized to the Renilla luciferase values to assess effects on translation. (C) Sample Western blots immunoblotted for NS5A and GAPDH (loading control) are shown. SGR–Feo–JFH-1 cells were treated with DCV, NPPB, IAA-94, or DIDS for 48 h. (D) SGR–Feo–JFH-1 cells were treated with the indicated Cl^−^ blockers; 48 h posttreatment, they were fixed with methanol and permeabilized in 50% methanol-acetone. NS5A was visualized via labeling with sheep anti-NS5A antisera followed by staining with Alexa Fluor 488-conjugated secondary antibodies. Representative confocal images are shown. (E) SGR-Feo-Con1 cells stably expressing a luciferase subgenomic replicon (genotype 1b) were treated with the Cl^−^ channel inhibitors, and luciferase activity was assessed as described for panel A. Error bars represent the SEM of results of stimulations from three independent experiments. **, significant difference from the value for the untreated control (*P* < 0.05). NS, no differences at the 0.05 significance level. (F) Naive Huh7 and SGR-Neo-JFH-1 cells were seeded into 12-well plates and treated for the indicated times with 10 μM NPPB and then were loaded with 5 mM 6-methoxy-quinolyl acetoethyl ester (MQAE) in Dulbecco modified Eagle medium (DMEM) for 1 h at 37°C. Following incubation, cells were washed three times with DMEM and fluorescent images immediately acquired using the IncuCyte Zoom live-cell imager. Mean fluorescence per cell was calculated from a minimum of three independent experiments performed in triplicate using IncuCyte Zoom live-cell imager software.

At the hepatocyte plasma membrane, Cl^−^ channels are absolutely required for cell volume control and apoptosis regulation (9, 10). Intracellularly, Cl^−^ transport across organelle membranes is involved in endosomal, lysosomal, and Golgi apparatus acidification (20, 21). We assessed several well-characterized Cl^−^ channel blockers, including 5-nitro-2-3-phenylpropylamino benzoic acid (NPPB), indyanyloxyacetic acid 94 (IAA-94), and diisothiocyanostilbene-2,20-disulfonic acid (DIDS) for their effects on SGR–Feo–JFH-1 replication. Figure 2A shows that NPPB and IAA-94 significantly inhibited HCV genome replication (10 μM, 57.7% ± 8% inhibition, and 100 μM, 62% ± 3% inhibition, respectively) and reduced NS5A expression, as assessed by Western blotting (Fig. 2C) and indirect immunofluorescence analysis (Fig. 2D). At these inhibitory concentrations, NPPB and IAA-94 did not affect cell viability (Fig. 2A, gray bars) or HCV internal ribosome entry site (IRES)-mediated translation (Fig. 2B), suggesting a specific inhibition of virus genome replication. The inhibitory effects of NPPB and IAA-94 were also confirmed in a genotype 1B-derived replicon (SGR-Feo-Con1) (63% ± 23% and 56% ± 27% inhibition, respectively), suggesting these effects to be conserved across genotypes (Fig. 2E). Surprisingly, DIDS, a well-characterized broadly acting inhibitor of anion exchangers, did not inhibit SGR replication (Fig. 2A to E). We reasoned that HCV genome replication is thus dependent on the function of an NPPB- and IAA-94-sensitive, DIDS-insensitive Cl^−^ channel. Several Cl^−^ channels with this pharmacological profile have been reported (22). Given these effects, we proceeded to examine Cl^−^ homeostasis during the HCV replication cycle. For this, we used the fluorescent indicator *N*-ethoxycarbonylmethyl-6-methoxy-quinolinium bromide (MQAE), a dye quenched by enhanced Cl^−^ influx and the subsequent increase in intracellular chloride concentration [Cl^−^]_i_. Figure 2F demonstrates that SGR–Feo–JFH-1-harboring cells display a 42% increase in [Cl^−^]_i_ compared to the level in parental Huh7 cells (gray bars), consistent with an enhanced basally active Cl^−^ inward conductance. The [Cl^−^]_i_ increase could be suppressed by prolonged treatment with NPPB (10 μM, 24 h), which reduced [Cl^−^]_i_ to levels comparable to those in untreated Huh7 cells (Fig. 2F, white bars). Taken together, these data confirm that SGR–Feo–JFH-1 cells possess enhanced transepithelial Cl^−^ transport through an NPPB-sensitive Cl^−^ channel.

**FIG 3:**
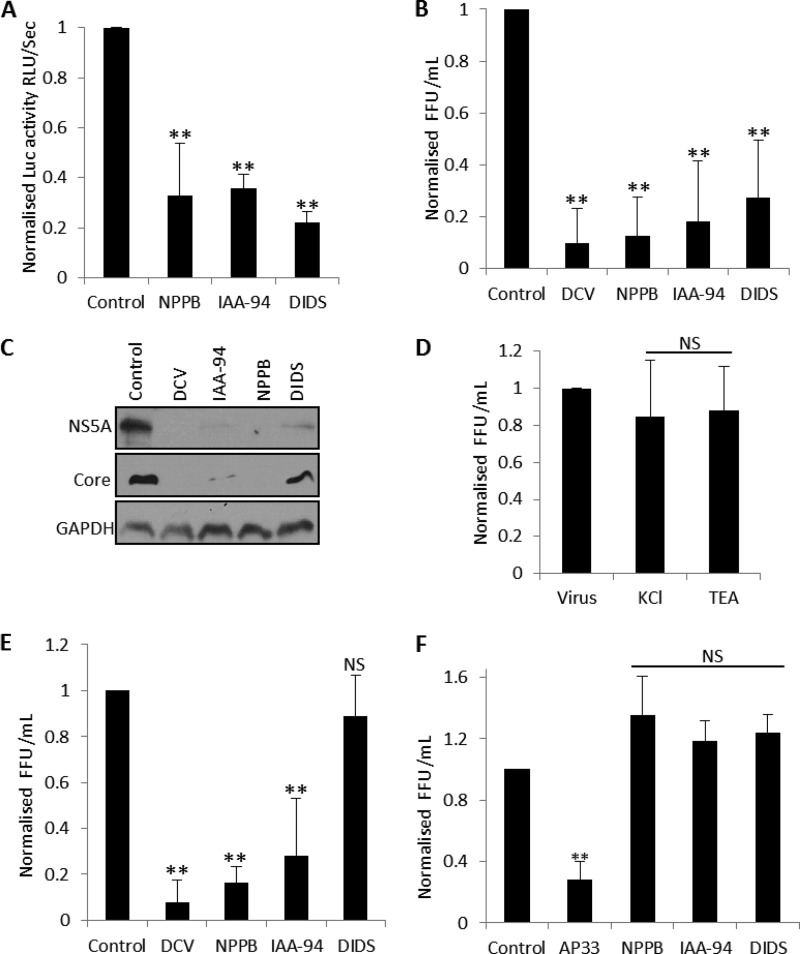
Assessment of the role of Cl^−^ channels during HCV infection. (A) Huh7 cells were pretreated with the indicated inhibitors for 45 min and infected with J6/JFH-1 RLuc virus at an MOI of 0.5 in the presence of each compound for 48 h. Cells were lysed and the levels of luciferase assayed as described for Fig. 1. (B) JFH-1 supernatants were produced by electroporation of cells with JFH-1 RNA and collection of the media from repeated passages for 2 weeks. Virus inoculum was clarified by centrifugation and added to Huh7 cells (5 × 10^4^ cells in six-well plates) in the presence of the indicated compounds. Cells were washed 24 h postinfection and were treated with medium plus compound for a further 48 h. Virus supernatants were collected and titrated onto Huh7 cells to assess numbers of focus-forming units (FFU)/ml. (C) Sample Western blots from the experiment discussed in panel B immunoblotted for NS5A, core, and GAPDH (loading control) are shown. (D) Experiments were performed as described for panel B in the presence of KCl and TEA at concentrations assessed in the experiment illustrated in Fig. 1. (E) Huh7 cells were infected with JFH-1 supernatants for 24 h. Cells were washed and replaced with medium plus compound for a further 48 h. Virus supernatants were collected, and virus production was assessed by focus-forming assays as described for panel B. (F) Compounds were mixed with virus inoculum in DMEM for 1 h and Huh7 cells infected at 37°C for 3 h. Cells were washed three times in phosphate-buffered saline (PBS) to remove unbound virus, and virus production was assessed 48 postinfection by focus-forming assays of virus supernatants. AP33 was included in these assays to inhibit HCV entry (50 mg/ml). All results were calculated relative to values for the untreated controls. **, significant difference from control value (*P* < 0.05); NS, no differences at the 0.05 significance level.

Since SGR–Feo–JFH-1-harboring cells express only the HCV nonstructural proteins NS3 to NS5B, it was important to determine whether Cl^−^ channel inhibition suppressed HCV replication in the context of virus-infected cells (23). We initially used a monocistronic full-length HCV chimeric genotype 2a virus, J6/JFH1, which is fully infectious in cell culture and expresses Renilla luciferase, herein termed J6/JFH-1 RLuc (24). Assays were performed by virus infection (multiplicity of infection [MOI] of 0.5) in the presence of each channel modulator, and luciferase expression was analyzed 48 h postinfection (p.i.). Figure 3A shows that NPPB and IAA-94 treatment significantly decreased J6/JFH-1 RLuc activity (67% ± 20% and 63% ± 5% inhibition, respectively) confirming a dependence on Cl^−^ influx during the virus life cycle. When these assays were performed in the presence of DIDS (100 μM), J6/JFH-1 RLuc activity also decreased by 77% ± 4% at concentrations that did not affect SGR–Feo–JFH-1 replication (Fig. 3A). To verify these data, we directly infected Huh7 cells with full-length JFH-1 virus (25) in the presence of each Cl^−^ inhibitor and measured the production of infectious virions by focus-forming assay. As shown in Fig. 3B, virus yields were significantly lower in IAA-94-, NPPB-, and DIDS-treated cells (87% ± 14%, 81% ± 23%, and 72% ± 22% inhibition, respectively). This was paralleled by a decrease of both NS5A and core protein expression in virus lysates as assessed by Western blot analysis (Fig. 3C). No effects on JFH-1 virus production were observed when TEA or KCl was assessed in these assays (Fig. 3D). We subsequently performed time-of-addition focus reduction assays using JFH-1 virus inoculum to assess the effects of DIDS over the time course of HCV infection. Cells were treated with each inhibitor 24 h p.i., and virus production was assessed 72 h p.i. Figure 3E shows that DCV, NPPB, and IAA-94 reduced JFH-1 virus production when added postinfection (92% ± 9%, 81% ± 23%, and 72% ± 22% inhibition, respectively), consistent with a block of HCV replication. DIDS however, failed to reduce virus production relative to that in the untreated wells, consistent with a lack of inhibition of HCV replication. To further determine which steps of the HCV life cycle are impaired by DIDS, we examined the effects of each Cl^−^ channel inhibitor on virus entry by adding them to JFH-1 inoculum during the initial 3 h of virus infection (26). The HCV-neutralizing mouse monoclonal E2 antibody AP33, a characterized inhibitor of HCV entry, was included in these assays for verification (27). Figure 3F shows that, while AP33 (50 μg/ml) inhibited HCV entry by 72% ± 11%, IAA-94, NPPB, and DIDS did not impede viral entry. These observations suggest that a DIDS-sensitive Cl^−^ channel can inhibit early postentry virion trafficking and/or early replication events but does not inhibit virus entry or replication following the establishment of infection.

**FIG 4:**
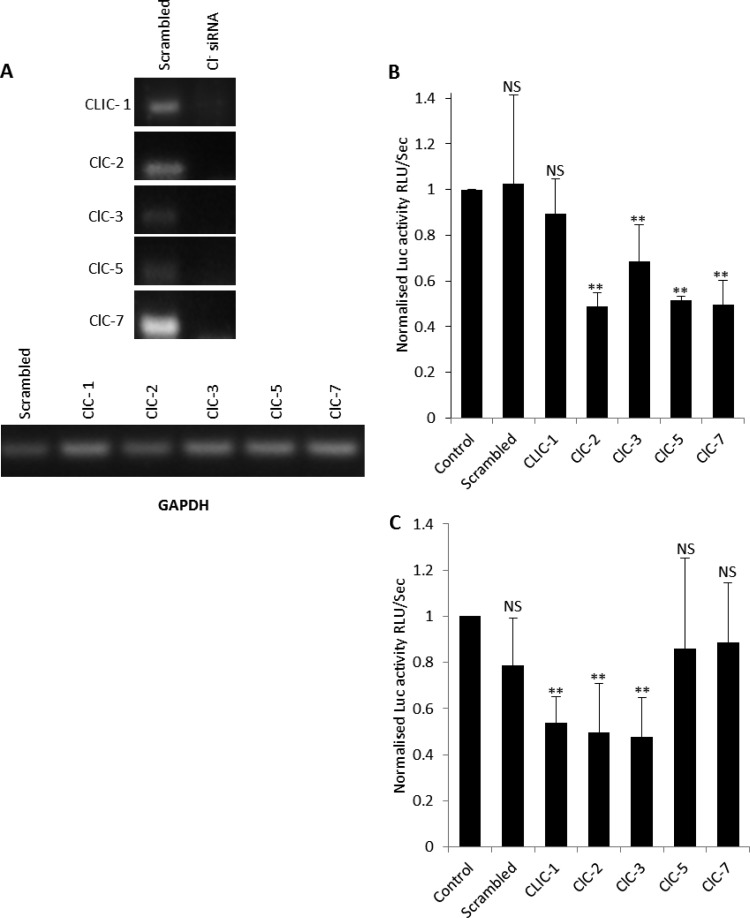
Assessment of the Cl^−^ channel(s) required for HCV replication. (A) Expression of CLIC-1, ClC-2, ClC-3, ClC-5, and ClC-7 was ablated by transfection of 75 pmol siRNA, as demonstrated by RT-PCR using whole-cell RNA extracts from SGR–Feo–JFH-1 cells. The siRNA had minimal impact on levels of GAPDH. (B) SGR–Feo–JFH-1 cells were treated with Cl^−^ channel siRNA or control siRNA, and luciferase expression was assessed as described for Fig. 1. Results were calculated relative to values for the transfection reagent-only controls. Error bars represent the SEM from three independent experiments. **, significant difference from the control value (*P* < 0.05); NS, no differences at the 0.05 significance level. (C) Huh7 cells were treated with Cl^−^ channel siRNA or control siRNA for 48 h and infected with J6/JFH-1 RLuc virus at an MOI of 0.5 for 48 h. Cells were lysed and the levels of luciferase assayed as described for Fig. 1. Error bars represent the SEM from four independent experiments. Values are normalized to those for the transfection controls. **, significant difference from value for the untreated control (*P* < 0.05). NS, no differences at the 0.05 significance level.

Given these data, we investigated the molecular identity of the Cl^−^ channel(s) required during the HCV life cycle. To date, nearly 40 different genes that, when expressed, increase Cl^−^ conductance have been cloned. These include the Cl^−^ intracellular-channel (CLIC) proteins cyclic AMP (cAMP) (CFTR)-, calcium (CaCC)-, voltage-activated Cl^−^ channels and Cl^−^/H^+^ exchangers (CLCs) as well as ligand-gated Cl^−^ channels [GABA(A), GABA(C), and glycine]. In hepatocytes, CLIC-1, ClC-2, ClC-3, ClC-5, and ClC-7 are expressed (9). We confirmed this by reverse transcription-PCR (RT-PCR) analysis (primer sequences are available upon request) and silenced this expression through small interfering RNA (siRNA) transfection (Fig. 4A). Figure 4B shows that ClC-2, ClC-3, ClC-5, and ClC-7 silencing significantly suppressed SGR–Feo–JFH-1 replication (52% ± 6%, 31% ± 16%, 48% ± 2%, and 50% ± 10% inhibition of luciferase activity, respectively). CLIC-1 knockdown displayed no discernible effects. Since some of these CLC channels and transporters are sensitive to NPPB and IAA-94; this confirmed the importance of Cl^−^ influx during HCV replication.

We finally investigated the effect of Cl^−^ channel silencing on J6/JFH-1 RLuc virus infection. Consistently with what occurred with SGR–Feo–JFH-1, silencing of ClC-2 and ClC-3 inhibited luciferase expression by 50% ± 21% and 52% ± 17%, respectively, confirming their requirement for virus replication. However, when ClC-5 and ClC-7 were silenced, no effects on J6/JFH-1 RLuc luciferase activity were observed despite their inhibitory effects on SGR–Feo–JFH-1 replication. Conversely, silencing of CLIC-1 decreased J6/JFH-1 RLuc luciferase activity by 46% ± 11% (Fig. 4C) despite a lack of effect on the replication of SGR–Feo–JFH-1 (Fig. 4B).

It is interesting to address what might be the molecular mechanisms underpinning the differential effects of CLIC-1, ClC-5, and ClC-7 silencing. While data on the biological role of CLIC-1 are limited, its activity has been shown to be required for the regulation of endosomal/lysosomal pH (9, 28). This may explain our observations, since the acidic late endosome/lysosome pH is crucial for induction of HCV glycoprotein (E1/E2) membrane fusion during early HCV postentry events to allow HCV genome release (29). The fact that ClC-5 and -7 are dispensable for J6/JFH-1 RLuc infectivity suggests that their function may be compensated for by HCV core-NS2 expression. ClC-5 is a known 2Cl^−^/H^+^ exchanger rather than a Cl^−^ channel, the function of which is to control endosomal acidification (30, 31). The HCV viroporin p7 is thought to form cationic intracellular channels that promote a global loss of organelle acidity (32, 33). p7 activity may thus prevent the buildup of an excess positive charge in specific organelles, a principle typically achieved by the import of Cl^−^ via anion transporters, including ClC-5.

Considering our findings together, we have confirmed the role of several Cl^−^ channel proteins during the HCV life cycle. Of note, we have identified ClC-2 and ClC-3, whose activities are required during HCV replication. Endosomal acidification and [Cl^−^]_i_ accumulation are significantly impaired in hepatocytes from ClC-2/3 knockout mice (20), and fractionation studies have suggested that these channels reside in early/late endosomes (34). The organization, composition, and functions of membrane structures induced by positive-strand RNA viruses remain largely ill defined but are generally accepted to require endosome integrity to recruit endosomal host cell factors and concentrate virus proteins to produce viral factories. Here, for the first time, we implicate host cell Cl^−^ influx through CLC channels/transporters during this process. The challenge will now be to define the specific virus-host interactions that require Cl^−^ channel functionality.
